# Context-Dependent Energetics of Loop Extensions in a Family of Tandem-Repeat Proteins

**DOI:** 10.1016/j.bpj.2018.03.038

**Published:** 2018-06-07

**Authors:** Albert Perez-Riba, Alan R. Lowe, Ewan R.G. Main, Laura S. Itzhaki

**Affiliations:** 1 Department of Pharmacology, University of Cambridge, Cambridge, United Kingdom; 2 London Centre for Nanotechnology, London, United Kingdom; 3 Structural & Molecular Biology, University College London, London, United Kingdom; 4 Department of Biological Sciences, Birkbeck College, University of London, London, United Kingdom; 5 School of Biological and Chemical Sciences, Queen Mary University of London, London, United Kingdom

## Abstract

Consensus-designed tetratricopeptide repeat proteins are highly stable, modular proteins that are strikingly amenable to rational engineering. They therefore have tremendous potential as building blocks for biomaterials and biomedicine. Here, we explore the possibility of extending the loops between repeats to enable further diversification, and we investigate how this modification affects stability and folding cooperativity. We find that extending a single loop by up to 25 residues does not disrupt the overall protein structure, but, strikingly, the effect on stability is highly context-dependent: in a two-repeat array, destabilization is relatively small and can be accounted for purely in entropic terms, whereas extending a loop in the middle of a large array is much more costly because of weakening of the interaction between the repeats. Our findings provide important and, to our knowledge, new insights that increase our understanding of the structure, folding, and function of natural repeat proteins and the design of artificial repeat proteins in biotechnology.

## Introduction

Tandem-repeat arrays are one of the most common protein architectures. Their high frequency is considered to be a result of DNA replication slippage and recombination events (1, 2). The *α*-solenoids are one large family composed of such tandem-repeat arrays. Their repeats comprise between 12 and 45 amino acids that form pairs of antiparallel *α*-helices. Examples include ankyrin repeats, armadillo repeats, HEAT repeats (huntingtin, elongation factor 3, protein phosphatase 2A subunit, and the yeast kinase TOR1), and tetratricopeptide repeats (TPRs) (3, 4, 5, 6). They function in mediating protein-protein interactions by providing extended surfaces for molecular recognition. Moreover, the modularity of their architectures has allowed the design of ultrastable consensus repeat proteins by selecting the most conserved residues in each family (7, 8, 9, 10, 11).

In contrast with globular proteins, repeat proteins have quasi-one-dimensional (1D) structures that are stabilized exclusively by interactions between residues close in primary sequence. Despite the lack of sequence-distant contacts, repeat proteins are able to fold in a cooperative manner. The cooperativity arises because of the mismatch between the intrinsically unstable repeats and the highly stabilizing inter-repeat interfaces (12). Repeat-protein folding can be modeled using 1D Ising formalism (13), which assumes that each repeat is either folded or unfolded, and that this state is determined by both the intrinsic repeat stability (*ΔG*_i_) and energetic coupling between the nearest neighbors, also referred to as the interface stability (*ΔG*_ij_). The simplest expression of the 1D Ising model, the homopolymer model, assumes single values of intrinsic and interfacial stabilities, and it has been shown to be valid for proteins comprising tandem arrays of identical repeats. One of the most important implications of this description of repeat protein folding is that the stability of the protein should scale linearly with the number of repeating units, referred to as the “additive rule” of the 1D Ising model:

ΔGD−N=nΔGi+(n−1)ΔGij,

where *n* is the number of repeating units (12, 13).

The folding of natural repeat proteins has been characterized both experimentally and in silico (14, 15, 16, 17, 18, 19, 20, 21, 22). The best-studied consensus-designed repeat proteins are the consensus ankyrin repeats (referred to as DARPins (8) or CARPs (7)) and consensus tetratricopeptide repeats (CTPRs) (13, 23). Although both have repeat units composed of pairs of antiparallel *α*-helices, they are structurally and energetically quite different. CARPs/DARPins are stabilized by a much larger interfacial term than the CTPRs. This can be attributed in part to the long semistructured loops of the former that have extensive hydrogen-bonding networks (24, 25). CTPRs, in contrast, have very short (four-residue) loops that are involved in a more limited, though still significant, number of stabilizing interactions (24, 25).

The structural simplicity of consensus-designed repeat proteins makes them popular systems to engineer for biotechnology purposes (10, 26, 27, 28, 29). Two significant outputs from these studies are the use of repeat proteins as building blocks for self-assembly systems and as alternatives to antibodies. In such systems, an avenue for further functionalization would be the extension of the loops between repeats to enable additional materials diversification. To this end, we created a series of 15 CTPR proteins that contained different numbers of repeats of different sequences. Into two of these proteins (CTPR2 and CTPR6) we engineered a loop between two adjacent repeats with a poly-GS linker of variable length between 10 and 25 residues. The loop-extension proteins, together with the other proteins within the series, were assayed using equilibrium denaturation experiments and globally analyzed using a heteropolymer Ising model. This global analysis allowed us both to determine the energetic contributions of nonidentical repeat units and to dissect the contributions from the intrinsic stability of each repeat and each interface between repeats. The results show that extending a single inter-repeat loop by up to 25 amino acids can be tolerated within the overall native structure. Moreover, although increasing the length of the inter-repeat loop weakens the nearest-neighbor cooperativity, it does not completely abolish it. Importantly, therefore, our results demonstrate that CTPR arrays are amenable to further functionalization through both large and small loop insertions. Strikingly, we find that the loss of stability associated with loop insertion is highly context-dependent: when a loop is inserted into a two-repeat array, the destabilization incurred is much smaller than the same loop inserted between the two central repeats of a six-repeat array. These results indicate that loop insertion destabilizes through both the entropic cost of loop closure and also the decoupling of the adjacent repeat modules.

In summary, our study provides important and, to our knowledge, new insights into the TPR proteins, a family with over 500,000 sequences in which long inter-repeat loops are often observed (30). Our results show that the insertion of a long loop between repeat motifs weakens the inter-repeat interface, which could cause the repeats to decouple, thereby stabilizing partly folded states. Such decoupling would enable loop-containing proteins to display enhanced conformational dynamics and/or mechanical flexibility. These properties may regulate the biological functions of natural repeat proteins and should be considered when used as an avenue for functionalization of artificial repeat proteins for biotechnological applications.

## Materials and Methods

### Construction of tandem-repeat genes from individual repeat sequences

#### CTPRn, CTPR-YD, and CTPR2-loop constructs

All constructs were commercially synthesized by GeneArt Invitrogen (Carlsbad, CA). Each construct was generated with a BamHI and a *Hin*dIII site for subcloning into pRSET for His-tag purification.

#### CTPRa2 construct

The tandem repeat arrays of two repeats was constructed by the concatemerization of two individual CTPRa motifs using BamHI and BglII sites (31). Briefly, a single CTPR (CTPRa1) was purchased as a “gBlock” oligo (Fig. S1) and inserted into the multicloning site of the vector pRSET B between the BamHI and *Hin*dIII restriction sites (ThermoFisher Scientific, Waltham, MA). An oligo consisting of the CTPRa1 “gBlock” was then polynucleotide-chain-reaction (PCR)-amplified using primers complementary to the T7 promoter sites on each side of the multicloning site of pRSET B. This PCR product and the CTPRa1 gene in the pRSET B vector were then digested with BamHI/*Hin*dIII and BglII/*Hin*dIII restriction enzymes, respectively. The two digested products could then ligate to form a CTPRa2 gene (as the BamH1 and BglII sites leave compatible ligation ends). The ligation of BamHI and BglII leaves an Arg and a Ser after the Pro at position 31 of the CTPR sequence. This results in a DPRS loop in the CTPRa2 (i.e., two-repeat array) (32). This process can be repeated as many times as required to generate CTPRa arrays of different lengths.

#### CTPR6-YD-loop constructs

Loop extensions of different length were added to the C-terminus of CTPR3n templates at the DNA-level by whole-plasmid round-the-horn PCR (33). This method enables large insertions to be made in a plasmid. Primers are designed so that they anneal back to back on the plasmid, with the desired insertion on the 5′-end of one primer (or separated onto both primers for large inserts).

### Protein purification

The pRSET B (His-tagged) constructs were transformed into chemically competent *Escherichia coli* C41 cells by heat shock and plated on LB-Amp plates (LB medium, MP Biomedicals; Agar, MP Biomedicals). Colonies were grown in 2xYT media (MP Biomedicals, Santa Ana, California) containing ampicillin (50 *μ*g/mL) at 37°C, 220 rotations per minute, until the optical density at 600 nm reached 0.6. Cultures were then induced with isopropyl β-D-1-thiogalactopyranoside (IPTG) (0.5 mM) for 16–20 h at 20°C. Cells were pelleted by centrifugation at 3000 × *g* (4°C, 10 min) and resuspended in lysis buffer (10 mM sodium phosphate pH 7.4, 150 mM NaCl, one tablet of SIGMAFAST protease inhibitor cocktail (Sigma-Aldrich, St. Louis, Missouri) (EDTA-free per 100 mL of solution), and lysed on an Emulsiflex C5 homogenizer (Avestin, Mannheim, Germany) at 15,000 psi. Cell debris was pelleted by centrifugation at 15,000 × *g* at 4°C for 45 min. Ni-NTA beads, 50% bed volume (GE Healthcare, Little Chalfont, UK) (5 mL), were washed once with phosphate buffer (10 mM sodium phosphate (pH 7.4), 150 mM NaCl) before binding the supernatant from the cell lysate for 1 h at 4°C in batch. The beads were washed three times with phosphate buffer (40 mL) containing 30 mM of imidazole to prevent nonspecific interaction of lysate proteins with the beads. Protein was eluted using phosphate buffer with 300 mM Imidazole and purified by size-exclusion gel filtration using a HiLoad 16/60 SuperdexG75 column (GE Life-Science, Little Chalfont, UK) preequilibrated in phosphate buffer (10 mM sodium phosphate (pH 7.4), 150 mM NaCl) and proteins separated in isocratic conditions. Purity was checked by NuPage protein gel (Invitrogen), and pure protein fractions were pooled. Purified protein was flash-frozen and stored at −80°C until further use. Concentrations were determined by absorbance at 280 nm using a calculated extinction coefficient (ExPASy ProtParam) (34) for each variant. Protein molecular weight and purity was confirmed using mass spectrometry (MALDI) (Mass Spectrometry Facility, Department of Chemistry, or PNAC, Department of Biochemistry).

### Circular dichroism spectroscopy

All circular dichroism (CD) measurements were made under the same configuration on a Chirascan CD spectrometer (Applied Photophysics, Leatherhead, UK) in 1-mm-pathlength Precision Cells (110-QS; Hellma Analytics, Müllheim, Germany) at 25°C. All protein samples (at 5–20 *μ*M concentration) were prepared in 50 mM sodium phosphate buffer (pH 6.8), 150 mM NaCl, and the CD spectrum was measured between 200 and 280 nm wavelengths using a 1-nm bandwidth unless specified otherwise. Measurements were taken at 1-nm intervals and were collected every 0.5 s; each reading was repeated between three and five times and the data averaged.

### Equilibrium denaturation monitored by fluorescence spectroscopy

High-throughput equilibrium denaturation experiments were carried out as previously described (35). Briefly, solutions were dispensed into Corning 96-well, half-area, black polystyrene plates (CLS3993; Corning, NY) with a Microlab ML510B dispenser (Hamilton, Reno, NV). All plate measurements were carried on a CLARIOstar Plate Reader (BMG LABTECH, Offenburg, Germany) with a tryptophan-detection set consisting of three filters, an excitation of 280–10 nm (275–285 nm), a dichroic PL325 nm, and an emission at 360–20 nm (350–370 nm) at 25°C. Protein concentrations were 0.3–1 *μ*M. For each protein, three sets of serial dilutions were plated consecutively. Plates were covered with Corning 96-well Microplate Aluminum Sealing Tape (Corning) to prevent evaporation, shaken for 30 s with the CLARIOstar double orbital shaking option, and incubated at 25°C for 1 h. The temperature was set at 25°C for the duration of the experiment.

### Equilibrium denaturation monitored by CD

Aliquots of GdmHCl (300 *μ*L) were prepared by dispensing the appropriate volume of stock solution of GdmHCl (7 M) in buffer (50 mM sodium phosphate buffer (pH 6.8), 150 mM NaCl) and sodium phosphate buffer (or otherwise indicated) using a Hamilton Microlab ML510B (Hamilton). Samples were equilibrated at 25°C for 2 h. The *α*-helicity was monitored by ellipticity at 222 nm. Results were plotted using GraphPad Prism (GraphPad Software, San Diego, CA).

### Equilibrium denaturation data analysis

Data were analyzed in two different ways as follows: they were either analyzed with a two-state model (36) or with a heteropolymer Ising model (12). Analysis of the data with the heteropolymer Ising model is described below. In the case of two-state model analysis, the protein chemical denaturations were fitted directly using Eq. 1:

(1) λobs=αN+βN[D]+(αD+βD[D])×exp[mD−N([D]−[D]50%)]RT1+exp[mD−N([D]−[D]50%)],

where *λ*_obs_ is the observed signal, *α*_N_ and *α*_D_ are the intercepts, *β*_N_ and *β*_D_ are the slopes of the baselines at the low (N) and high (D) denaturant concentrations, [D]_50%_ is the midpoint of unfolding, [D] is the concentration of denaturant, and *m*_D-N_ is a constant that is related to the increase in solvent exposure of the protein upon unfolding (37).

Equation 1 is based on a two-state model of denaturation in which only the native and the denatured states are populated and assumes that the signal of the native state, *λ*_N_, and the denatured state, *λ*_D_, are linearly dependent on the denaturant concentration (*λ*_N_ = *α*_N_ + *β*_N_[D], *λ*_D_ = *α*_D_ + *β*_D_[D]); for a detailed derivation, see (36). Values for [D]_50%_ and *m*_D-N_ are obtained with their standard errors. The free energy of unfolding in water can then be calculated using Eq. 2:

(2) $$\Delta {\text{G}}_{D-N}^{H_{2}O}=m_{D-N}\times {\left[D\right]}_{50\%},$$

where $\Delta {\text{G}}_{D-N}^{H_{2}O}$ is the free energy of unfolding in water, $m_{D-N}$ is the *m*-value, and ${\left[D\right]}_{50\%}$ is the equilibrium midpoint.

### Heteropolymer Ising model

For the Ising analysis, each equilibrium denaturation curve was individually converted to fraction unfolded $\left(\lambda_{\text{U}}\right)$ using Eq. 3:

(3) $$\lambda_{\text{U}}=\frac{\lambda_{\text{obs}}-\left(\alpha_{\text{N}}+\beta_{\text{N}}\left[D\right]\right)}{\left(\alpha_{\text{D}}-\alpha_{\text{N}}\right)+\left(\beta_{\text{D}}-\beta_{\text{N}}\right)\left[D\right]},$$

where *α*_D_/*α*_N_ are the *y*-intercept values of the denatured/native baselines and *β*_D_/*β*_N_ are the slopes of the denatured/native baselines.

After normalization, all of the curves were globally fitted to a heteropolymer Ising model using the PyFolding package (38). We constructed the 1D heteropolymer Ising model using a matrix formulation as previously described (12). Briefly, the model comprises a 1D linear series of equilibrium constants. These account for the intrinsic folding stability (*ΔG*_*i*_) and the interfacial energy (*ΔG*_*i−1,i*_) for each repeated unit in a nearest-neighbor TPR array. The intrinsic stability of the repeating unit has an associated coefficient (*m*) to represent its sensitivity to the external stimulus—in this case, chemical denaturant.

In previous studies on CTPR proteins, the repeating Ising unit used has been at the level of individual helices within each array (13, 32, 39). Here, the CTPR series were fitted to both 1) different repeating units of individual helices and 2) different repeating units of TPR motifs. The fits showed that the model with different repeating unit of TPR motifs gives better agreement to the experimental data. This is most likely due to the nature of the input protein series used, i.e., the input proteins differ in number of TPR motifs as opposed to one with differing numbers of helices. Thus, asymmetry of CTPR proteins was modeled via unique sets of parameters to represent a “standard” CTPR motif ($\Delta G_{i}^{CTPR}$, $\Delta G_{i-1,i}^{CTPR}$, and $m^{CTPR}$), a CTPR motif with the D to Y mutation ($\Delta G_{i}^{CTPR-Y91D}$, $\Delta G_{i-1,i}^{CTPR-Y91D}$, and $m^{CTPR-Y91D}$), and inserted single loops with the CTPR motif proceeding it ($\Delta G_{i}^{loop-CTPR}$, $\Delta G_{i-1,\;i}^{Loop-CTPR}$, and $m^{Loop-CTPR}$). The *m* parameters ($m^{CTPR}$, $m^{CTPR-Y91D}$, and $m^{Loop-CTPR}$) gave a denaturant dependence to the intrinsic stabilities. The expressions defining the equilibrium constants (Eqs. 4 and 5) and the protein partition function, *q*(*n*) are given below (Eq. 6):

(4) $$\kappa_{i}={\text{e}}^{\left[-\left(\Delta G_{i}-mx\right)/RT\right]}.$$

and

(5) $$\tau_{i-1,i}={\text{e}}^{\left[-\Delta G_{i-1,i}/RT\right]},$$

where *ΔG*_*i*_ is the free energy of folding for the domain at position *i*, with denaturant sensitivity *m* and at denaturant concentration *x*. *ΔG*_*i−1,i*_ is the free energy for the interface between domains at positions *i* − 1 and *i*. *R* is the gas constant, and *T* is experimental temperature in Kelvin.

The full partition function of the protein with *n* repeat motifs is given by Eq. 6:

(6) $$q\left(n\right)=\begin{array}{cc} \left[0\;1\right] & \left[\begin{array}{cc} \kappa_{1}\tau_{-1} & 1\\ \kappa_{1} & 1 \end{array}\right] \end{array}\cdots \left[\begin{array}{cc} \kappa_{n}\tau_{n-1} & 1\\ \kappa_{n} & 1 \end{array}\right]\left[\begin{array}{c} 1\\ 1 \end{array}\right].$$

This defines the fully folded state. The model allows for fitting of separate parameters (*κ* and *τ*, thus $\Delta G_{i}$, $\Delta G_{i-1,i}\;$, and *m*) to describe behavior of the various repeat motif units by globally fitting to data for degenerate CTPR protein compositions.

The fraction folded, $\lambda_{\text{F}}$, is then simply defined as the sum of the subpartition functions divided by the number of terms (repeat motifs) multiplied by the full partition function (Eqs. 7 and 8):

(7) $$q\left(i\right)=\begin{array}{cc} {}[0 & 1] \end{array}\cdots \left[\begin{array}{cc} \kappa_{i}\tau_{i-1} & 0\\ \kappa_{i} & 0 \end{array}\right]\cdots \left[\begin{array}{c} 1\\ 1 \end{array}\right]$$

and

(8) $$\lambda_{\text{F}}=\;\frac{1}{nq\left(n\right)}\sum_{i=0}^{n}q\left(i\right).$$

From the fitted variables, the stability of any CTPR ensemble or part thereof $\left(\Delta G_{0\to j}^{H20}\right)$ can be calculated by adding energy terms (Eq. 9):

(9) $$\Delta G_{0\to j}^{H20}=\;n\Delta Gi\;+\;\left(n-1\right)\Delta Gi,j\;=\;-RT\;\ln\;\kappa^{n}\tau^{\left(n-1\right)},$$

where $\Delta G_{0\to j}^{H20}$ is the free energy of folding in water for a protein with *j* repeat motifs, *n* is the number of folded repeat motifs in each protein, *ΔG*_*i*_ is the free energy of folding for the motif at position *i*, and *ΔG*_*i−1,i*_ is the free energy for the interface between motifs at positions *i* − 1 and *i*.

### Stopped-flow fluorescence

Aliquots of guanidinium hydrochloride (GdmHCl) were prepared by dispensing the appropriate volume of stock solution of GdmHCl in sodium phosphate buffer (50 mM sodium phosphate buffer (pH 6.8), 150 mM NaCl) using a Hamilton Microlab ML510B dispenser (Hamilton). For each protein, two aliquots (3 mL) were prepared to a final concentration of 10 *μ*M of protein. One aliquot was fully folded in sodium phosphate buffer (or low concentrations of GdmHCl), and the other denatured in 6 M GdmHCl. Samples were equilibrated at 10 or 25°C for 2 h. The proteins and the GdmHCl solutions were mixed at a 1:5 ratio. An excitation wavelength of 280 nm was used, and the emission was measured using a 330-nm cutoff filter. Unfolded protein was refolded by rapid mixing with increasing concentrations of GdmHCl up to the denaturation midpoint as defined by equilibrium denaturation. Folded protein was unfolded by rapid mixing with increasing concentrations of GdmHCl above the equilibrium denaturation midpoint. Multiple traces were acquired at each GdmHCl concentration, averaged and then fitted to a single exponential or a double exponential in GraphPad Prism.

Chevron plots that showed nonlinear folding and/or unfolding arms were fitted using a broad transition state barrier model originally described by Oliveberg and coworkers (40). Nevertheless, the fit was simply qualitative, as the refolding rates of these CTPR proteins are faster than the limit of detection of our instrument:

(10) $$\text{ln}k_{obs}=\ln\left(k_{f}^{H_{2}O}exp(-m_{k_{f}}\left[denaturant\right]\right)+\;\text{exp}\left(-m_{k_{f}}^{*}{\left[denaturant\right]}^{2}\right)+k_{u}^{H_{2}O}+\text{exp}\left(m_{k_{u}}\left[denaturant\right]\right)+\text{exp}\left(m_{k_{u}}^{*}{\left[denaturant\right]}^{2}\right)).$$

### Statistical analysis

All measurements were performed in triplicate unless indicated otherwise, and the errors for the two-state fits are the standard errors of the mean. The errors from $\Delta G_{D-N}^{H_{2}O}$ calculation were propagated from standard errors of the mean. Errors of the fitted variables by the 1D heteropolymer Ising model were determined by calculating a covariance matrix from the Jacobian matrix after a subsequent least-squares minimization of the fit. Errors in $\Delta G_{0\to 1}^{H20}$ were propagated from the errors obtained from the fitted variables.

### Data availability

iPython Jupyter notebooks of the heteropolymer Ising model analysis are included as Supporting Materials and Methods. All data is available upon request. To create the figures in the study, the fitting results from PyFolding were exported as CSV files and plotted using the program Prism (GraphPad Software).

## Results

### Design of consensus-repeat modules and loop extensions

**Figure 1:**
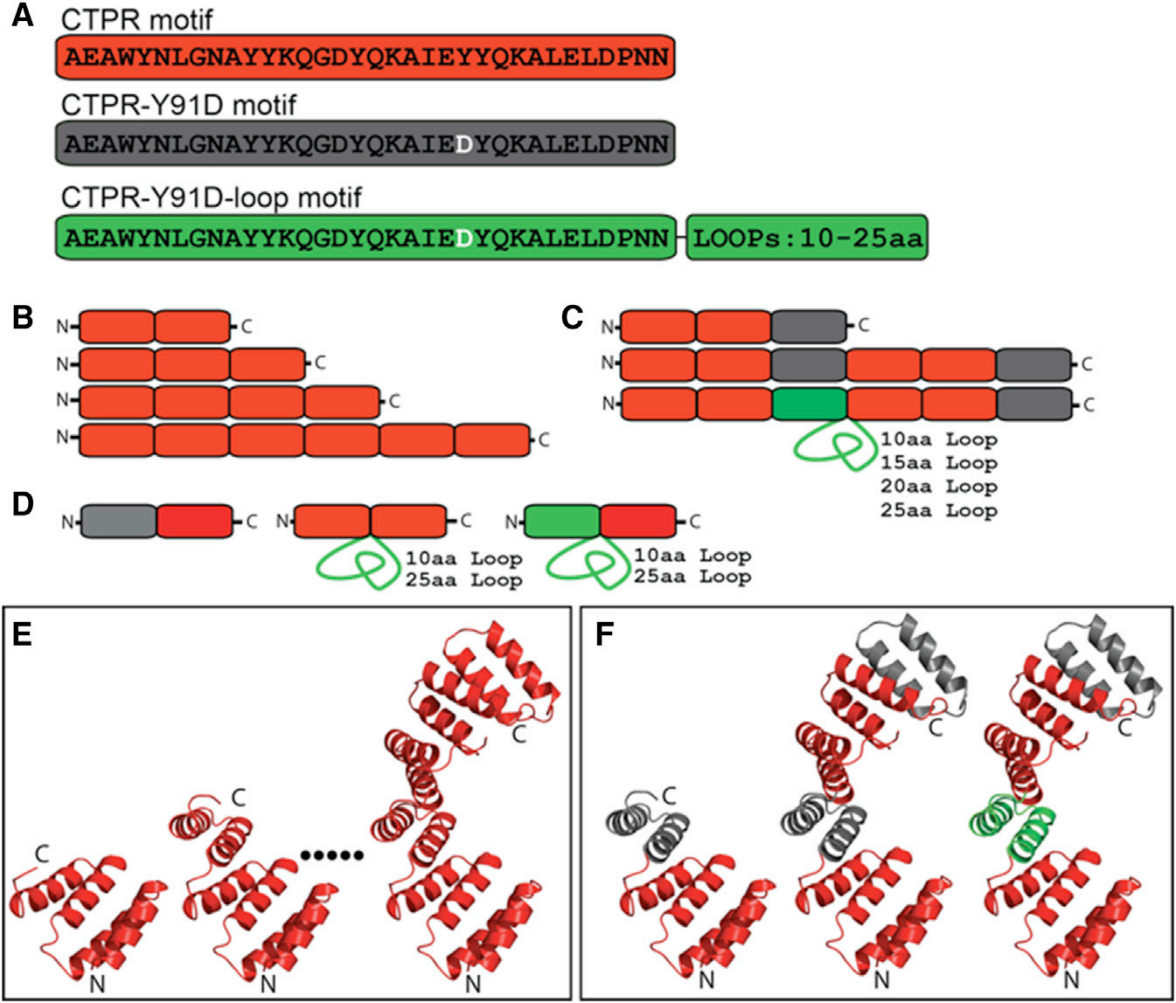
Sequences, topologies, and modeled structures of the 15 proteins used in this study. (*A*) The repeating TPR motif sequences used (each repeat contains two *α*-helices) are as follows: CTPRn (*red*), CTPR-YD (*gray*), and CTPR-YD-loop (*green*). (*B*) The topology of the CTPR series of four proteins containing only the “CTPR” motif (CTPR2, CTPR3, CTPR4, and CTPR6) (9) is shown. Repeats are colored as per (*A*) to show that all proteins in this series contain only the CTPR sequence. (*C*) The topology of the CTPR series containing “CTPR,” “CTPR-Y91D,” and “CTPR-Y91D-loop” motifs (CTPR3-YD, CTPR6-YD, CTPR6-YD-loop10, CTPR6-YD-loop15, etc.) is shown. Repeats are colored as per (*A*) to show where the loop-containing and YD-containing repeats occur. (*D*) The topology of the CTPR2 series containing “CTPRn,” “CTPR-Y91D,” and “CTPR-Y91D-loop” motifs (CTPRa2 and CTPR2 with either a 10-residue or a 25-residue loop between the two repeats and a version of them with the Y-to-D point mutation) is shown. Repeats are colored as per (*A*) to show where the loop-containing and YD-containing repeats occur. (*E*) A ribbon representation of the atomic structures of CTPR2, CTPR3, and CTPR6 based on the crystal structure 2HYI (56) is shown. The dots represent the fact that this series also includes CTPR4 (data not shown). Repeats are colored as per (*A*) to show that all proteins contain only the CTPRn sequence. (*F*) A ribbon representation of the atomic structures of CTPR3-YD, CTPR6-YD, and CTPR6-YD-loop proteins based on crystal structure 2HYI (56) is shown. Repeats are colored as per (*A*) to show that, for example, CTPR3-YD is composed of two CTPR repeats and a C-terminal CTPR-YD repeat. In the representation of the CTPR6-YD-loop proteins, the CTPR-YD-loop motif is located in repeat 3 (*green*). The loops were inserted after the third repeat (*green*) and before the fourth repeat (*red*). The sequences for all proteins are found in Table S1. To see this figure in color, go online.

In this study, we constructed 15 CTPR proteins that contain different numbers of repeats with two consensus-repeat sequences differing by a point mutation and a single-loop insertion of different lengths (shown schematically in Fig. 1). Comparison of the biophysical characteristics of all these different CTPR constructs enabled us to delineate the effects of loop insertion and of size of loop inserted versus the effects of point mutation. The 15 CTPR proteins consisted of the following: 1) a CTPR3 module (comprising three CTPR motifs), as studied previously by Regan and colleagues and referred to here as CTPR3-YD. This CTPR sequence contained a single-point mutation, Y91D, in the third repeat relative to other published CTPR sequences (41); 2) a six-repeat series built from two CTPR3-YD modules with either a native loop between the two modules (CTPR6-YD) or a poly-GS loop of differing length between 10 and 25 residues inserted between the two CTPR3-YD modules (CTPR6-YD-loop10, CTPR6-YD-loop15, etc.). The poly-GS loop contained a thrombin cleavage site that allowed us to demonstrate that the loop is solvent-accessible (see Fig. S4); 3) a series of four proteins (CTPR2, CTPR3, CTPR4, and CTPR6) comprising between two and four repeats of the original consensus sequence in (9); and 4) a two-repeat series comprising CTPRa2 and CTPR2 with either a 10-residue or a 25-residue loop between the two repeats and versions of them with the Y-to-D point mutation. To simplify the analysis, all of the proteins lack the C-terminal so-called “solvating” helix used in some previous studies. All expressed in *E. coli* in a soluble form and eluted as single monomer-sized peaks when subjected to size-exclusion chromatography.

### Comparison of the CTPR, CTPR-YD, and CTPR-YD-loop constructs: loop extension compromises the thermodynamic stability and cooperativity but not the overall native structure

**Figure 2:**
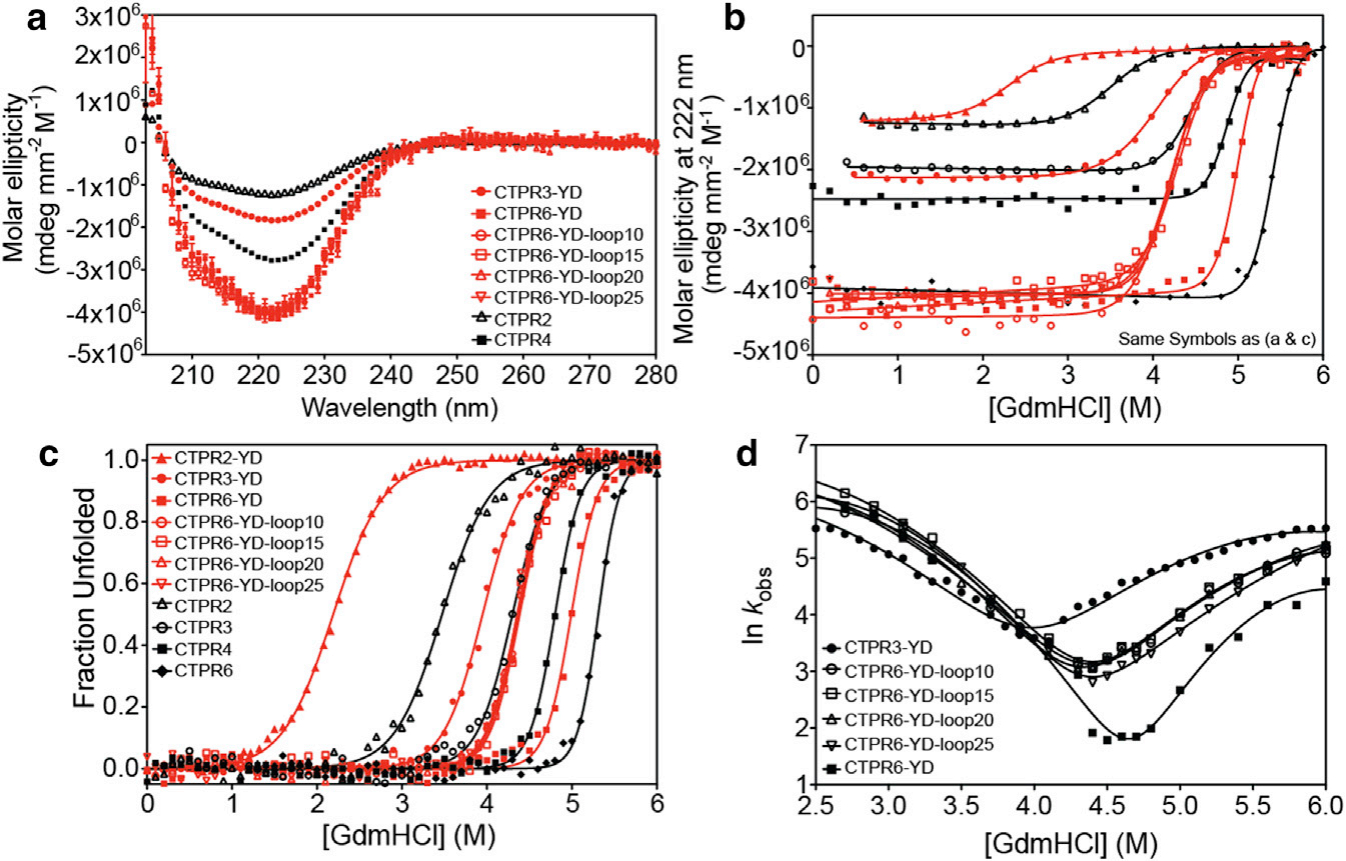
Biophysical analysis and comparison of the CTPR, CTPR-Y91D, and CTPR-Y91D-loop series of proteins. (*a*) Far-UV CD spectra, (*b* and *c*) averaged equilibrium denaturation curves monitored by (*b*) CD at 222 nm and converted to molar ellipticity and (*c*) fluorescence converted to fraction unfolded for ease of comparison, and (*d*) chevron plots are shown. The denaturation curves are fitted to a two-state model. The chevron plots are fitted to a two-state model in which folding and unfolding reaction proceed via a broad transition-state model. Measurements were performed at 25°C in 50 mM sodium phosphate buffer (pH 6.8), 150 mM NaCl. To see this figure in color, go online.

To determine whether loop insertion radically alters the secondary structure of the native state, for example, by unfolding repeats or decoupling sections of the CTPR array into independently folding units, far-ultraviolet (UV) CD spectra were recorded and thermal/chemical denaturations performed. Far-UV CD spectra show that the CTPR6-YD loop-extension constructs have the same *α*-helical content as CTPR6-YD (Fig. 2 *a*). Moreover, the CTPR6-YD-loop series showed very high melting temperatures, similar to that of CTPR6-YD (Fig. S2). Thus, a single loop extension of up to 25 residues does not compromise the native structure of CTPR6-YD protein.

Next, chemical denaturation experiments were performed by monitoring both tryptophan fluorescence (there is a tryptophan residue in each repeat) and CD (monitored at 222 nm). Initially, all curves were fitted to a two-state equation to give the midpoints of unfolding (*D*_50%_), *m*-values, and free energies of unfolding (Table 1). Fig. 2, *b* and *c* shows a comparison of the denaturation curves of the CTPR6-YD-loop proteins with those of the CTPR series and CTPR-YD series, from which a number of features and trends are apparent.

**Table 1:** Parameters Obtained by Fitting the Equilibrium Denaturation Data to a Two-State Model for the CTPR, CTPR-YD, and the CTPR6-YD-Loop Proteins Series

Experiment/Protein	*D*_50%_ (M)	*m*-Value (kcal mol^−1^ M^−1^)	$\Delta G_{D-N}^{H_{2}O}$ (kcal mol^−1^)
Equilibrium Denaturation Monitored by Fluorescence			

CTPR2	3.53 ± 0.01	2.09 ± 0.04	−7.4 ± 0.1
CTPR3	4.30 ± 0.02	2.8 ± 0.2	−12.0 ± 0.9
CTPR4	4.80 ± 0.01	4.0 ± 0.3	−19.2 ± 0.9
CTPR6	5.30 ± 0.03	4.8 ± 0.2	−25.5 ± 1.1
CTPR2-YD	2.28 ± 0.01	2.1 ± 0.04	−4.8 ± 0.1
CTPR3-YD	3.93 ± 0.02	2.9 ± 0.2	−11.4 ± 0.8
CTPR6-YD-loop10	4.35 ± 0.01	3.4 ± 0.1	−14.9 ± 0.6
CTPR6-YD-loop15	4.32 ± 0.02	3.1 ± 0.3	−13.4 ± 1.1
CTPR6-YD-loop20	4.37 ± 0.02	3.4 ± 0.3	−14.9 ± 1.3
CTPR6-YD-loop25	4.38 ± 0.02	3.1 ± 0.2	−13.6 ± 0.9
CTPR6-YD	4.99 ± 0.03	4.5 ± 0.5	−22.5 ± 2.3

Equilibrium Denaturation Monitored by CD			

CTPR2	3.50 ± 0.01	2.2 ± 0.03	−7.6 ± 0.1
CTPR3	4.46 ± 0.01	3.3 ± 0.04	−10.3 ± 0.1
CTPR4	4.85 ± 0.01	4.8 ± 0.1	−23.3 ± 0.5
CTPR6	5.41 ± 0.01	4.9 ± 0.1	−26.5 ± 0.5
CTPR2-YD	2.32 ± 0.02	1.8 ± 0.1	−4.2 ± 0.1
CTPR3-YD	3.96 ± 0.01	2.31 ± 0.03	−9.6 ± 0.1
CTPR6-YD-loop10	4.19 ± 0.01	2.69 ± 0.06	−11.3 ± 0.3
CTPR6-YD-loop15	4.24 ± 0.01	2.64 ± 0.04	−11.2 ± 0.2
CTPR6-YD-loop20	4.21 ± 0.01	3.08 ± 0.04	−12.9 ± 0.2
CTPR6-YD-loop25	4.20 ± 0.01	2.88 ± 0.04	−12.1 ± 0.2
CTPR6-YD	4.97 ± 0.01	4.3 ± 0.1	−21.3 ± 0.4

All measurements were performed in triplicate, and the errors listed are the SE of the mean. The $\Delta G_{D-N}^{H_{2}O}$ for the loop-extension proteins are apparent values only, as their low *m*-values indicate that the unfolding transitions are not fully cooperative.

First, each chemical denaturation curve, whether monitored by CD or fluorescence, showed a single unfolding transition. Moreover, there is good agreement between denaturation curves monitored by CD and by fluorescence. This result indicates that denaturation occurs via concurrent loss of native secondary and tertiary structures. Importantly, the native pretransition baselines of the CD-monitored denaturations were essentially flat. Thus, the single-loop and single-point-mutation-containing proteins do not partially unfold before the major transition.

Second, the chemical denaturations of the four loop variants overlay and give the same *D*_50%_ and *m*-values when fitted to a two-state equation. Significantly, these values are lower than those of the parent protein, CTPR6-YD, yet higher than “half” of it (CTPR3-YD). The inserted loop, therefore, appears to cause a loss in stability and cooperativity, and this effect is independent of the length of the loop. However, because the CTPR6-YD-loop variants have significantly higher *D*_50%_ and *m*-values than those of CTPR3-Y91D, the repeats must be folded as a CTPR6 unit rather than exist as two fully uncoupled CTPR3-YD halves. The two-state fits of the data indicate an apparent loss in stability of 7.5 kcal mol^−1^ upon loop extension (Table 1).

### Un/folding kinetics of the loop-extension constructs show that loss of thermodynamic stability is mainly through increased rates of unfolding and TPR motifs are not uncoupled

The unfolding and refolding kinetics of the proteins were measured using stopped-flow fluorescence. The refolding traces for all proteins were fitted to the sum of two exponential phases, the faster of which constituted ∼80–95% of the overall amplitude (Fig. S3). The smaller, slower phase could be the result of proline isomerization, as there is a proline residue in each CTPR module (at the end of the second helix). The refolding traces at GdmHCl concentrations below 2.5 M were too fast to be fitted accurately. The unfolding traces were fitted to a single exponential phase (Fig. S3).

Both unfolding and refolding kinetics are shown in Fig. 2 *d* as chevron plots. These show that all proteins exhibit curvature in both the refolding arm and the unfolding arm. Therefore, although the kinetics is more complex than a simple two-state transition, two effects of loop extension are readily apparent. First, the loop-extension proteins have rate constants for unfolding that lie between those of the three-repeat and six-repeat arrays, CTPR3-Y91D and CTPR6-Y91D. Second, loop extensions have only a small effect on the refolding rates. Thus, the kinetics show that the major effect of the loop extension is to destabilize the native state via increased unfolding rates. Moreover, the intermediate nature of the loop constructs’ chevron plots corroborates the equilibrium finding that the loops do not completely uncouple the six-repeat protein into two CTPR3-YD units.

### Delineating the effects of loop extension on stability and cooperativity using 1D heteropolymer Ising model analysis

**Figure 3:**
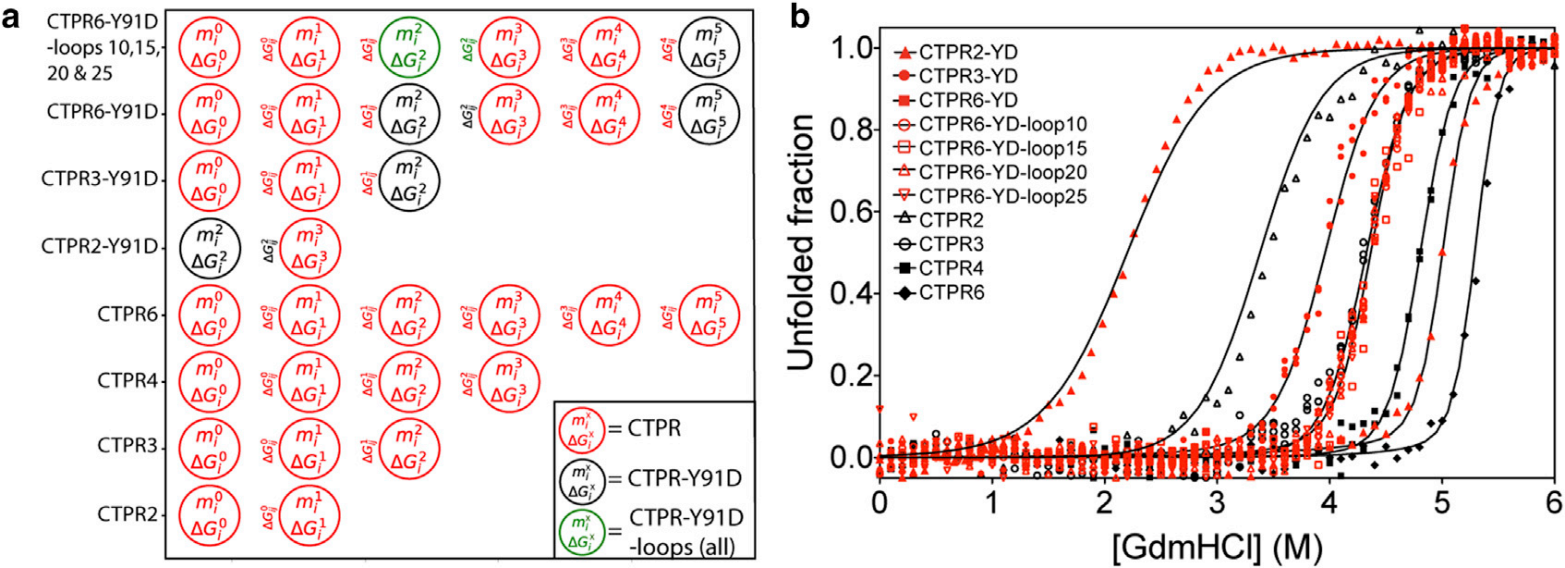
Equilibrium denaturation curves for the CTPR, CTPR-YD, and CTPR-YD-loop proteins fitted globally to a 1D heteropolymer Ising model. (*a*) Topologies used for each protein when fit to the Heteropolymer Ising model are shown as follows: CTPR repeat (*red*), the CTPR-YD repeat (*black*), and the CTPR-YD-loop repeat (*green*). (*b*) Equilibrium denaturation curves for the CTPR, CTPR-YD, and CTPR-YD-loop proteins fitted globally to a 1-D Heteropolymer Ising model. The minimal unit of repetition was set as an individual helix-turn-helix-loop repeat. To see this figure in color, go online.

The above two-state fitting of the equilibrium denaturation data is only of limited, qualitative use, given that there is clearly evidence of deviation of the loop-extended protein from this simple model. Global Ising model analysis of repeat-protein denaturation curves has been shown to be an effective means of quantifying repeat-protein energetics, as it enables us to dissect the contribution that individual repeat units make (inter-repeat interfacial energy and intrinsic repeat stability) to the overall stability and cooperativity (11, 39, 42, 43). Here, we use a heteropolymer Ising model, as our TPR arrays are composed of nonidentical repeat motifs (Fig. 1; Supporting Material). We therefore globally fitted 27 denaturation curves of the following eleven proteins (the majority of which were performed in triplicate): the CTPR series (CTPR2, CTPR3, CTPR4, and CTPR6), the loop series (CTPR6-YD-loop10, CTPR6-YD-loop15, CTPR6-YD-loop20, and CTPR6-YD-loop25), and the mutant series (CTPR2-YD, CTPR3-YD, and CTPR6-YD) (Fig. 3), thereby determining the energetics of all three types of repeat units used (CTPR, CTPR-YD, and CTPR-YD-loop), for which the unit of repetition was defined as the whole TPR motif, i.e., helix-turn-helix-loop. As there was no significant length dependence of the stability of the CTPR6-YD-loop series, we fitted all of them with the same energetic terms. The denaturation curves were first converted to fraction unfolded (using Eq. 3), as the CD data showed that there was no pretransition unfolding of the proteins. The heteropolymer model was able to describe the equilibrium denaturation curves of these 11 proteins with a total of nine globally fitted parameters. These parameters are the intrinsic stability (*ΔG*_i_), the interfacial stability (*ΔG*_ij_), and the *m*-value (*m*_i_) for each of the three types of repeat units (CTPR, CTPR-YD, and CTPR-YD-loop). Fig. 3 show the high quality of the fits, and Table 2 summarizes the results.

**Table 2:** Values of Intrinsic and Interfacial Stabilities for the Three Different Repeat Units Analyzed Using the Heteropolymer Model

Repeat Type	*ΔG*_i_ (kcal mol^−1^)^a^	*ΔG*_ij_ (kcal mol^−1^)^a^	*m*_i_ (kcal mol^−1^ M^−1^)^a^	$\Delta G_{0\to 1}^{H20}$ ((kcal mol^−1^)^b^
CTPR	−0.59 ± 0.12	−6.08 ± 0.08	1.1 ± 0.7	−6.7 ± 0.2
CTPR-YD	2.53 ± 0.07	−5.60 ± 0.01	0.6 ± 0.3	−3.1 ± 0.2
CTPR-YD-loop	2.59 ± 0.03	−1.36 ± 0.03	0.38 ± 0.12	1.2 ± 0.1

Only the intrinsic stability term has a denaturant dependence (*m*_i_). Intrinsic stabilities are represented as *ΔG*_i_. Interfacial stabilities are represented as *ΔG*_ij_.

a Errors of the fitted variables were determined by calculating a covariance matrix from the Jacobian matrix after a subsequent least-squares minimization of the fit.

b $\Delta G_{0\to 1}^{H20}$ = $\Delta G_{i}^{H20}+\;$$\Delta G_{i-1,i}^{H20}$, i.e., the stability gained when a single repeat is added to a folded TPR ensemble. Errors in $\Delta G_{0\to 1}^{H20}$ were propagated from the errors obtained from the fitted variables.

The Ising model confirms the two-state analysis, showing that the CTPR-YD loop-containing repeat is the least stable, followed by the point-mutation-containing CTPR-YD repeat, with the CTPR repeat being the most stable. Furthermore, the Ising model analysis shows that the destabilizing effect of the point mutation is mostly localized to the intrinsic energy term, whereas the effect of loop extension was mostly localized to the interfacial energy term with little effect on the intrinsic energy term. Thus, the energetic effect of the loop insertion relative to that of the point mutation can be calculated as *ΔΔG =* $\Delta G_{0\to 1}^{H20}$ (CTPR variant 1) − $\Delta G_{0\to 1}^{H20}$ (CTPR variant 2), where $\Delta G_{0\to 1}^{H20}$ = $\Delta G_{i}^{H20}+\;$ $\Delta G_{i-1,i}^{H20}$. Table 3 summarizes the results and shows the effect of the point mutation (3.3 ± 0.3 kcal mol^−1^) compared with the loop (4.3 ± 0.4 kcal mol^−1^). This means that the loop value is four times the energetic cost of a 10-residue loop extension observed previously for globular proteins (1.1 kcal mol^−1^) as calculated by the Ising model and seven times as calculated by the two-state model (44). The difference between the two may be a result of partially folded intermediate states being taken into account in the Ising model.

**Table 3:** Energetic Costs of the YD Mutation and the Loop Extension, Calculated as the Changes in the Free Energy of Unfolding from a Heteropolymer Ising Model Fit

	*ΔΔG* (kcal mol^−1^)^a^
CTPR to CTPR-YD	+3.6 ± 0.3
CTPR to CTPR-YD-loop	+7.9 ± 0.2
CTPR to CTPR-loop	+4.3 ± 0.2

Free energy of unfolding is represented as *ΔΔG*.

a *ΔΔG =*$\Delta G_{0\to 1}^{H20}$ (CTPR variant 1) − $\Delta G_{0\to 1}^{H20}$ (CTPR variant 2), where $\Delta G_{0\to 1}^{H20}$ = $\Delta G_{i}^{H20}+\;$$\Delta G_{i-1,i}^{H20}$. Errors in *ΔΔG* were propagated from the errors in $\Delta G_{0\to 1}^{H20}$.

### Loop extension incurs only a small energetic cost in the context of a two-repeat array

**Figure 4:**
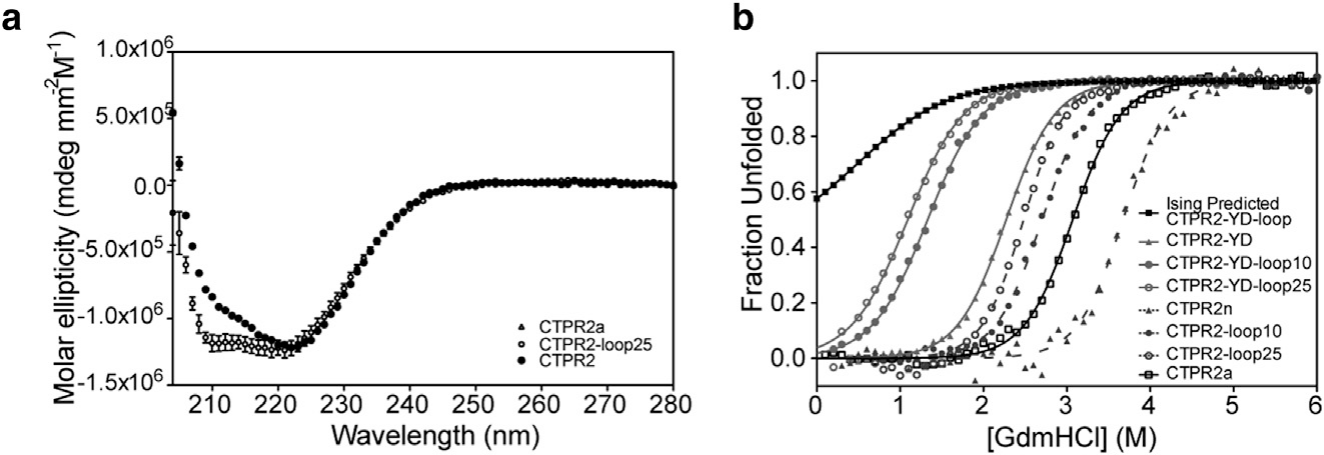
Effects of loop extension on the two-repeat CTPR array. (*a*) CD spectra of CTPRa2, CTPR2, and CTPR2-loop25 are given. (*b*) Equilibrium denaturation curves monitored by fluorescence (converted to fraction unfolded for comparison) for all CTPR2 variants (CTPR2, CTPRa2, loops, and YD series) and CTPR2-YD-loop predicted according to Ising behavior are given. The data are fitted to a two-state model. All measurements were performed at 25°C in 50 mM sodium phosphate buffer (pH 6.8), 150 mM NaCl.

The additivity rule of the Ising model allows us to predict the stability of a protein comprising any combination of CTPR, CTPR-YD and CTPR-loop units. The large stability loss of loop extension observed for the six-repeat protein would be predicted to render a two-repeat protein with the YD mutant (CTPR2-YD-loop) to be mostly unfolded and a two-repeat protein (CTPR2) to be very destabilized (see the predicted denaturation curve for CTPR2-YD-loop in Fig. 4 *b*). To test this prediction, we made four two-repeats proteins with and without the Y-to-D mutation and with loop extensions of 10 residues and 25 residues: CTPR2-YD-loop10 and CTPR2-YD-loop25, and CTPR2-loop10 and CTPR2-loop25. Previous reports on CTPR proteins have demonstrated how changing the amino-acid composition of the short loop between repeats has a small but significant effect on the interfacial stability. Specifically, changing the sequence from NN to RS results in a loss in stability of 1 kcal mol^−1^ because of differences in side-chain interactions upon mutation. This effect was found to follow the additivity rule of the Ising model (32). We do not know how the loop extension would affect these loop interactions, and therefore, we made an additional CTPR2 variant with the DPRS sequence (CTPRa2) for comparative purposes.

Fig. 4 *a* shows a comparison of the CD spectra of CTPR2-loop25 and CTPRa2. As CTPRs are all-helical proteins, they should show a double minimum in the CD spectrum at 208 and 222 nm. However, CTPR proteins do not have a pronounced 208-nm minimum (9, 23, 39). Interestingly, the spectrum of CTPR2-loop25 did show the double minimum expected for an *α*-helical protein. The similar 222-nm ellipticities of CTPR2-loop25 and CTPR2 indicate that loop extension does not compromise the overall structure of the protein.

Fig. 4 *b* shows a comparison between the experimentally observed denaturation curves of all the CTPR2 variant proteins (CTPR2, CTPRa2, loops and YD series) with the Ising-predicted denaturation curve based on the energetic terms obtained from the CTPR6 variants as discussed above. As can be seen, all of the two-repeat proteins had the same *m*-value within error, indicating that folding cooperativity is not perturbed by loop extension (Table 4). The stability loss because of the DPNN-to-DPRS mutation was 1 kcal mol^−1^, the same as the value obtained from the six-repeat data (and consistent with previous measurements (32)). However, the energetic cost of the loop extension in the two-repeat protein was ∼2.5-fold smaller than the value of 4.3 kcal mol^−1^ obtained from the heteropolymer model for the CTPR-loop in the six-repeat protein. It is also noteworthy that this energetic cost is length-dependent, unlike the length-independent effect of loop extension observed for the six-repeat array. Fersht and colleagues used the following polymer model to predict the entropic cost of a loop extension in a globular protein (45):

(11) $$\Delta \Delta G=\;-\text{T}\;\Delta \Delta S_{config.}=\;-\text{T}\left(-\frac{3}{2}\right)\text{R}ln\left(\frac{n+\delta n}{n}\right),$$

where *n* is the loop length and *δn* is the length of the extension. Accordingly, the entropic cost should be 1.1 kcal mol^−1^ for a 10-residue loop extension and 1.75 kcal mol^−1^ for a 25-residue loop extension. These values are much closer to those observed for the loop extensions in the two-repeat array (Table 5). As would be expected, globally fitting the CTPR2-YD-loop proteins together with the other series to the heteropolymer Ising model produced values that were not thermodynamically consistent with the data, further underlining the observation that loop extension in a CTPR2 array is not energetically equivalent to loop extension in a CTPR6 array.

**Table 4:** Fit of the Equilibrium Denaturation Data to a Two-State Model for the CTPR2 Proteins

Protein	D_50%_ (M)^a^	*m*-Value (kcal mol^−1^ M^−1^)^a^	$\Delta G_{D-N}^{H_{2}O}$ (kcal mol^−1^ M^−1^)^b^
CTPR2a	3.07 ± 0.02	2.09 ± 0.04	−6.4 ± 0.1
CTPR2n	3.53 ± 0.01	2.09 ± 0.04	−7.4 ± 0.1
CTPR2-YD	2.28 ± 0.01	2.09 ± 0.04	−4.8 ± 0.1
CTPR2-YD-loop10	1.43 ± 0.02	2.09 ± 0.04	−3.0 ± 0.1
CTPR2-YD-loop25	1.25 ± 0.02	2.09 ± 0.04	−2.6 ± 0.1
CTPR2n-loop10	2.71 ± 0.02	2.09 ± 0.04	−5.7 ± 0.1
CTPR2n-loop25	2.45 ± 0.02	2.09 ± 0.04	−5.1 ± 0.1

All measurements were performed in triplicate.

a Errors are the SEs of the mean.

b Errors were propagated from the errors obtained from the fitted variables.

**Table 5:** Energetic Cost of the Point Mutation and Loop Extensions in a Two-Repeats Array of CTPRs

	$\Delta \Delta G_{D-N}^{H_{2}O}$ (kcal mol^−1^)
Cost of YD mutation in CTPR2n	2.6 ± 0.1
Cost of RS loop instead of NN loop	1.0 ± 0.1
Cost of loop10 in CTPR2-YD	1.8 ± 0.1
Cost of loop25 in CTPR2-YD	2.2 ± 0.1
Cost of loop10 in CTPR2	1.7 ± 0.1
Cost of loop25 in CTPRn	2.3 ± 0.1
Theoretical entropic cost of a loop10^a^	1.1
Theoretical entropic cost of a loop25^a^	1.7

$\Delta \Delta G_{D-N}^{H_{2}O}$ values are calculated as the difference in $\Delta G_{D-N}^{H20}$ between the specified proteins. Errors were propagated from the errors obtained from the fitted variables.

a The theoretical entropic cost of both loop lengths.

## Discussion

Here, we have asked whether TPR proteins can be functionalized by extending the loops between repeats and how these structural alterations affect their folding. It is interesting to compare TPRs to ANK-repeat proteins in this respect (7, 13, 23, 43), as the major differences between them are the lengths of the helices and of the inter-repeat loops. ANK proteins have shorter helices that contribute less to stability than the longer TPR ones. However, the long semistructured inter-repeat loops in ANKs contribute to high overall stability through forming a network of stabilizing hydrogen bonds. This creates a large mismatch between intrinsic and interfacial stabilities, thereby resulting in highly cooperative folding (19).

The mismatch of intra- and inter-repeat stabilities is smaller in the TPRs (13, 39). The interfacial stability of the CTPRs is provided mainly by the hydrophobic packing between *α*-helical residues in adjacent repeats, with a smaller contribution from specific interactions of residues in the inter-repeat loop. Disruption of the loop contacts upon mutation of the NN sequence to RS decreases the overall stability of the repeat (∼1 kcal mol^−1^ per loop) (32). According to polymer theory, a loop extension of 10 residues should have a similar-sized energetic cost as the NN to RS mutation, with longer loops having greater entropic penalty (44). Consequently, we would expect that a loop-extended CTPR array should still be highly stable. However, what we observe is different from this prediction: a single loop extension introduced into the two middle repeats of a six-repeat array causes a much larger than expected and length-independent decrease in both stability and cooperativity. Strikingly, when the same loop is inserted into a two-repeat array, only a small and length-dependent loss of stability is observed, similar to that predicted by polymer theory. Moreover, there was no significant effect on the *m*-value, indicating that cooperativity of the two-repeat array is not compromised by the loop extension. In contrast, loop insertion in a six-repeat array lowered the both the *m*-value and *D*_50%_ and brought these values close to, but importantly, still larger than that of a three-repeat array.

The folding behavior of CTPR proteins is dependent on the number of repeats: CTPR2 has been described as the most two-state like, resembling a four-helix bundle (i.e., a globular protein) as much as a tandem-repeat array. Increasing the number of repeats in the array results in an increase in the overall stability of the protein because of the nearest-neighbor cooperativity between repeats and the mismatch between intrinsic and interfacial stability. The central repeats have been shown by hydrogen-deuterium exchange experiments to be the most highly protected from solvent and therefore the least likely to explore unfolded conformations (11, 46, 47). Moreover, the degree of protection increases with increasing number of repeats in the array, the trend breaking down only when the number of repeats in the array is sufficiently large for intermediates to be populated. We have shown that loop extension weakens the unfolding cooperativity of the array. We would therefore expect the loop-extended repeat to be much less protected from hydrogen-deuterium exchange than the consensus counterpart. TPRs (and ankyrin repeat proteins) have been shown to exhibit dynamic spring-like behavior in solution, whereby a spring constant can be used to define the frequency of the protein “breathing” (16, 48, 49, 50, 51); thus, the loss of nearest-neighbor cooperativity and stability induced by loop extension should manifest as an increase in dynamic properties at the loop-extended interface.

In conclusion, our study shows that the introduction of loops into CTPR arrays is context-dependent and can lead to a more dynamic and less stable CTPR protein array than expected. TPR proteins function as molecular scaffolds (52, 53, 54, 55), and long loops of 10 or more residues are commonly observed (30). The break in cooperativity, the population of intermediates, and the dynamic and mechanical consequences of a weakened inter-repeat interface may be important for their mechanism of action and/or regulation of binding partners. Importantly, we have shown that large inter-repeat loop extensions can nevertheless produce very stable and natively folded CTPR arrays. Although folding cooperativity is weakened, it is not completely destroyed. Thus, our study demonstrates that CTPR arrays are amenable to both large and small loop insertions ready to be exploited in various biotechnology and biomedical applications.

## Author Contributions

A.P.-R. and L.S.I. conceived and designed the experiments. A.P.-R. carried out the experiments. A.P.-R., E.R.G.M., and A.R.L. performed the data analysis. A.P.-R., L.S.I., E.R.G.M., and A.R.L. wrote the manuscript.

## Supporting Material

Document S1. Supporting Materials and Methods, Figs. S1–S4, and Table S1

Document S2. Article plus Supporting Material
