# Supplementary material for: Context-Dependent Energetics of Loop Extensions in a Family of Tandem-Repeat Proteins
Source: Biophys J. 2018 Jun 7;114(11):2552–62. doi: 10.1016/j.bpj.2018.03.038 (PMC6129472; doi:10.1016/j.bpj.2018.03.038)
Supplement: Document S1. Supporting Materials and Methods, Figs. S1–S4, and Table S1 [file mmc1.pdf]

Biophysical Journal, Volume *114*

Supplemental Information

Context-Dependent Energetics of Loop Extensions in a Family of Tandem-Repeat Proteins

Albert Perez-Riba, Alan R. Lowe, Ewan R.G. Main, and Laura S. Itzhaki

Supplemental information

Supplemental data items

Table S1. Amino acid sequences of the proteins used in this study. Related to Figure 1.

Proteins	Sequence of the CTPR motifs in each construct
CTPRn	$(AEAWYNLGNAYYKQGDYQKAIEYYQKALELDPNN)_n$
CTPRa	$(AEAWYNLGNAYYKQGDYQKAIEYYQKALELDPRS)_n$
CTPR2-loop10	AEAWYNLGNAYYKQGDYQKAIEYYQKALELDPNNGSLVPRGSRS<br>AEAWYNLGNAYYKQGDYQKAIEYYQKALELDPNN
CTPR2-loop25	AEAWYNLGNAYYKQGDYQKAIEYYQKALELDPNNSGGGGSGGLVPRGSGSGGGGSGRS<br>AEAWYNLGNAYYKQGDYQKAIEYYQKALELDPNN
CTPR2-YD	(AEAWYNLGNAYYKQGDYQKAIEDYQKALELDPNN<br>AEAWYNLGNAYYKQGDYQKAIEYYQKALELDPNN
CTPR2-YD-loop10	AEAWYNLGNAYYKQGDYQKAIEDYQKALELDPNN GSLVPRGSRS<br>AEAWYNLGNAYYKQGDYQKAIEYYQKALELDPNN
CTPR2-YD-loop25	AEAWYNLGNAYYKQGDYQKAIEDYQKALELDPNN SGGGGSGGLVPRGSGSGGGGSGRS<br>AEAWYNLGNAYYKQGDYQKAIEYYQKALELDPNN
CTPR3-YD	$(AEAWYNLGNAYYKQGDYQKAIEYYQKALELDPNN)_2$<br>AEAWYNLGNAYYKQGDYQKAIEDYQKALELDPNN
CTPR6-YD	$(AEAWYNLGNAYYKQGDYQKAIEYYQKALELDPNN)_2$<br>AEAWYNLGNAYYKQGDYQKAIEDYQKALELDPNN<br>$(AEAWYNLGNAYYKQGDYQKAIEYYQKALELDPNN)_2$<br>AEAWYNLGNAYYKQGDYQKAIEDYQKALELDPNN
CTPR6-YD-loop10	$(AEAWYNLGNAYYKQGDYQKAIEYYQKALELDPNN)_2$<br>AEAWYNLGNAYYKQGDYQKAIEDYQKALELDPNNGSLVPRGSRS<br>$(AEAWYNLGNAYYKQGDYQKAIEYYQKALELDPNN)_2$<br>AEAWYNLGNAYYKQGDYQKAIEDYQKALELDPNN
CTPR6-YD-loop15	$(AEAWYNLGNAYYKQGDYQKAIEYYQKALELDPNN)_2$<br>AEAWYNLGNAYYKQGDYQKAIEDYQKALELDPNNGSGGLVPRGSGSGRS<br>$(AEAWYNLGNAYYKQGDYQKAIEYYQKALELDPNN)_2$<br>AEAWYNLGNAYYKQGDYQKAIEDYQKALELDPNN
CTPR6-YD-loop20	$(AEAWYNLGNAYYKQGDYQKAIEYYQKALELDPNN)_2$<br>AEAWYNLGNAYYKQGDYQKAIEDYQKALELDPNNGSGGGGSLVPRGSGGGSGRS<br>$(AEAWYNLGNAYYKQGDYQKAIEYYQKALELDPNN)_2$<br>AEAWYNLGNAYYKQGDYQKAIEDYQKALELDPNN
CTPR6-YD-loop25	$(AEAWYNLGNAYYKQGDYQKAIEYYQKALELDPNN)_2$<br>AEAWYNLGNAYYKQGDYQKAIEDYQKALELDPNNGSGGGGSGGLVPRGSGSGGGGSGRS<br>$(AEAWYNLGNAYYKQGDYQKAIEYYQKALELDPNN)_2$<br>AEAWYNLGNAYYKQGDYQKAIEDYQKALELDPNN

The CTPR proteins used here contain the stabilising Gln-Lys mutation (green) (Cortajarena et al., 2011) instead of the original (Asp-Glu) consensus sequence (Main et al., 2003a). The mutation Y to D is present in the CTPR-YD and CTPR6-YD-loop series (purple). Both DPNN and DPRS (red) sequences are used in this work. The poly-GS sequences of variable length and containing a thrombin cleavage site (blue) were inserted after the consensus DPNN sequence into the inter-repeat loop of the two-repeat arrays (or between the third and fourth repeats of the six-repeat arrays).

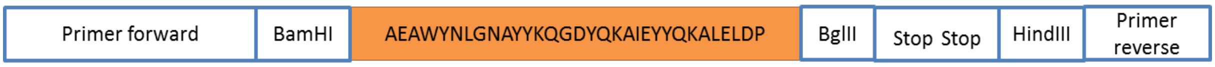

Figure S1. The gBlock oligo of a CTPRa1 (i. e. a single CTPRa consensus motif). The oligo contains the restriction sites, the stop codons and the annealing regions for PCR amplification.

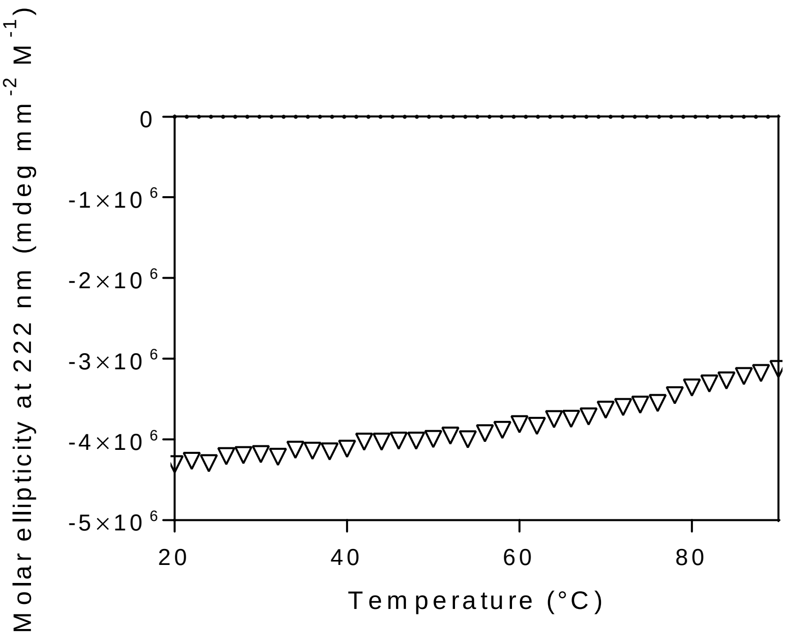

Figure S2. Partial thermal denaturation of CTPR6-YD-loop25 monitored by (b) CD at 222 nm and converted to molar ellipticity.

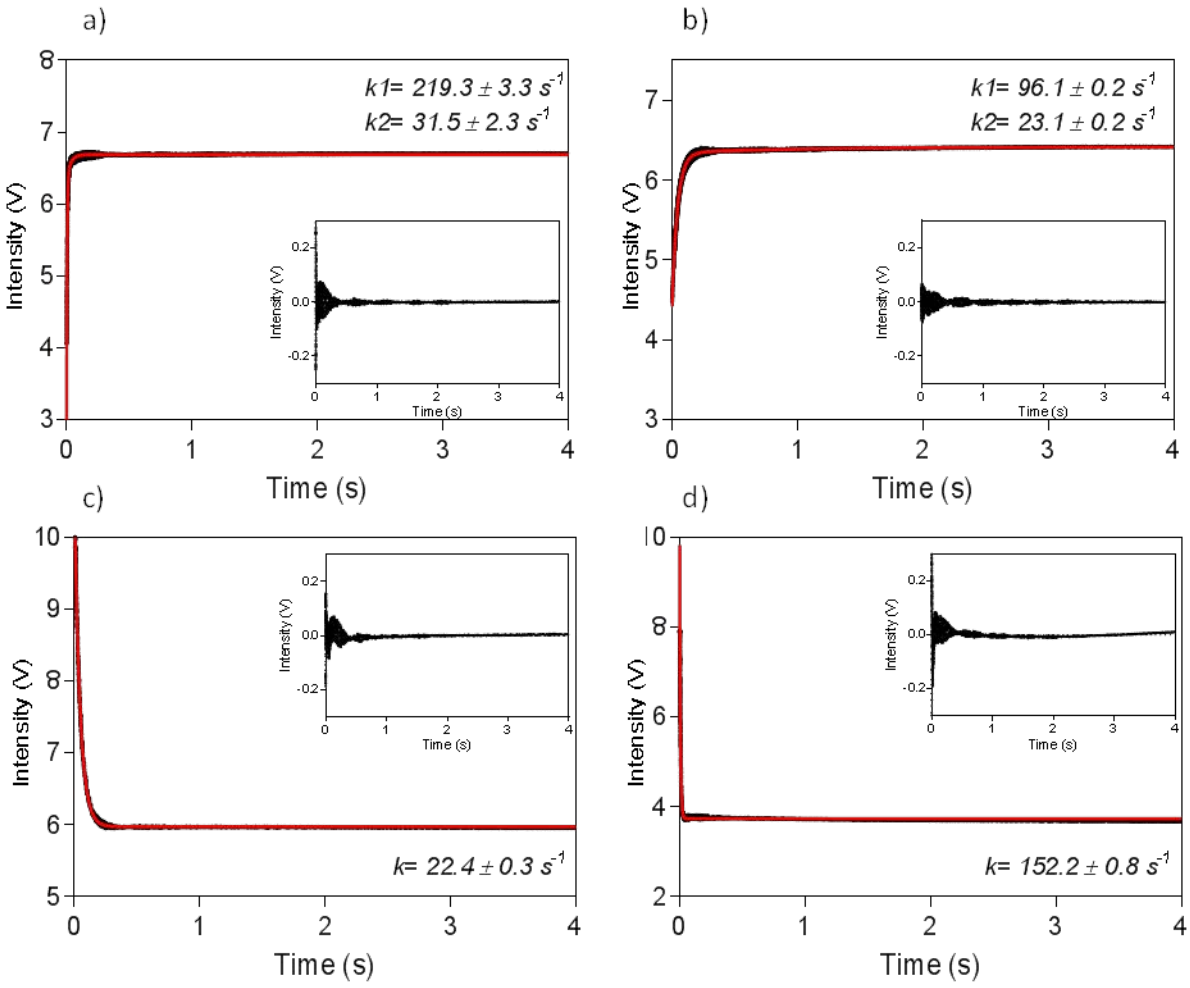

Figure S3. Representative kinetic traces monitored by stopped-flow fluorescence at 10°C for CTPR6sl10 in 50 mM sodium phosphate buffer pH 6.8, 150 mM NaCl. Double-exponential fitting for refolding at a final concentration of (a) 2 M GdmHCl and (b) 4 M GdmHCl resepectively. Single-exponential fitting for unfolding at a final concentration of (c) 4.5 M GdmHCl and (d) of 5.5 M GdmHCl. Residuals of the fit are shown in the inset plots. Residuals for unfolding at 5.5 M GdmHCl are shown without fitting to a sloping baseline to highlight the deviation of the fit observed.

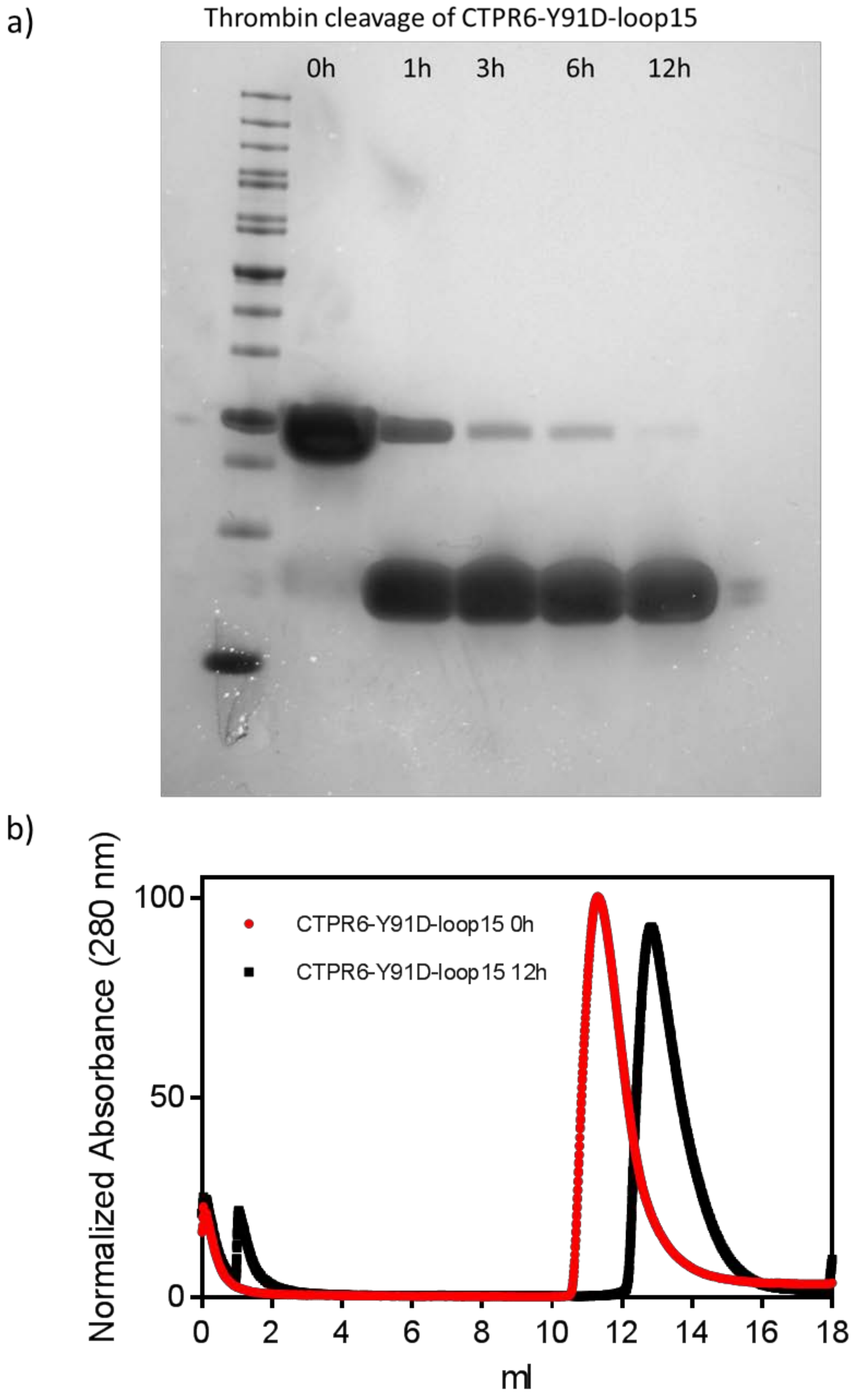

Figure S4. a) SDS-NuPAGE Bis-Tris gel showing different incubation times for thrombin cleavage of CTPR6-Y91D-loop15. b) Elution profiles by analytical filtration using a Superdex 200 column of the CTPR6-Y91D-loop15 protein (red) and the thrombin-cleaved CTPR6-Y91D-loop15 protein (black) monitored by absorbance at 280 nm.

Supplemental Experimental Procedures

Thrombin cleavage of the CTPR6sl protein

A Thrombin CleanCleave Kit (Sigma-Aldrich) was used to cleave CTPR6-Y91D-loop15 and also the CTPR6-Y91D-control. Proteins were analysed after different time with SDS NuPage protein gel (Invitrogen) (Fig. S4). Clearly, the cleavage is specific for the loop site. The reaction was found to require 12 h at room temperature for complete cleavage. These samples were analysed by analytical gel filtration, which showed that the repeat-interface does not remain associated once the covalent linkage has been lost (Fig. S4).
